# The revised international technical guidance on sexuality education - a powerful tool at an important crossroads for sexuality education

**DOI:** 10.1186/s12978-018-0629-x

**Published:** 2018-11-06

**Authors:** Joanna Herat, Marina Plesons, Chris Castle, Jenelle Babb, Venkatraman Chandra-Mouli

**Affiliations:** 1 UNESCO Section of Health and Education, Paris, France; 2 WHO Department of Reproductive Health and Research / Human Reproduction Programme, Geneva, Switzerland

**Keywords:** Comprehensive sexuality education, Adolescents, Sexual and reproductive health

## Abstract

In January 2018, UNESCO, together with UNAIDS, UNFPA, UNICEF, UN Women, and the WHO, completed the substantial technical and political process of updating the International Technical Guidance on Sexuality Education, thereby unifying a UN position on rationale, evidence, and guidance on designing and delivering comprehensive sexuality education (CSE). The revised Guidance builds on the original Guidance, with improvements and updates based on new evidence and good practice documented from across the globe. User-surveys and structured consultations with representatives from a wide range of fields and interest-groups informed and guided the revision process. The revised Guidance presents one, commonly agreed definition of CSE; enhances and expands its key concepts, topics and learning objectives; places a strengthened focus on gender and human rights; provides guidance on building support and planning the implementation of CSE programmes; and reflects the contribution of CSE to the realization of multiple Sustainable Development Goals (SDGs). With its unified voice, progressive position, and attention to key implementation challenges, the revised Guidance is a responsive, timely, and critically needed tool to advance towards a tipping point for the large-scale application of quality CSE.

## ᅟ

In January 2018, UNESCO, together with UNAIDS, UNFPA, UNICEF, UN Women, and the WHO completed the substantial technical and political process of updating the International Technical Guidance on Sexuality Education, thereby unifying a UN position on rationale, evidence, and guidance on designing and delivering comprehensive sexuality education (CSE) [1]. This achievement has resounding implications for the advancement of global development agendas and for the health and well-being of adolescents around the world.

## Why was the Guidance updated?

Since the original Guidance was published in 2009, the field of CSE has evolved rapidly in light of evidence from research and lessons learnt from the implementation of sexuality education programmes across diverse educational settings [2].

The evidence for the need to support children and adolescents’ knowledge and behaviours related to their health and well being remains compelling. In some parts of the world, two out of three girls reported having no idea what was happening to them when they began menstruating [3, 4]. In a number of countries, condom use at last high-risk sex in the previous year was less than 50% for young people aged 15–24 years [5]. In too many places around the world, sexual and reproductive health (SRH) issues and problems such as these are barriers for many learners – especially girls – to fulfilling their right to education. The associated poor health outcomes further compound and negatively impact their life opportunities and potential.

Meanwhile, the evidence for the need to support children and adolescents’ attitudes and norms related to their health and well being has strengthened. Worldwide, close to 50% of all girls aged 15–19 years believe a husband or partner is justified in hitting or beating his wife (or partner) under certain circumstances – if the wife argues with her husband, goes out without telling him, neglects the children, refuses to have sexual relations with him, or burns the food [6]. Where SRH issues are shaped by social and cultural attitudes and norms that entrench inequalities of gender and power, the transformative role of CSE in challenging such attitudes and norms adds value to the already important contribution made by CSE in building inclusive, sustainable societies.

CSE has been recognized as an important entry point for promoting adolescent health, which has gained global priority through the Sustainable Development Goals (SDGs) that call for attention to adolescents both as an end in itself and as a means to the end of overall health and well being of populations. Furthermore, the global health, education and development agendas and frameworks, in particular the 2030 Agenda for Sustainable Development, have shifted towards an increased recognition of the intrinsic links between education, health and well-being, gender equality and human rights.

## What is new in the revised Guidance?

**Fig. 1:**
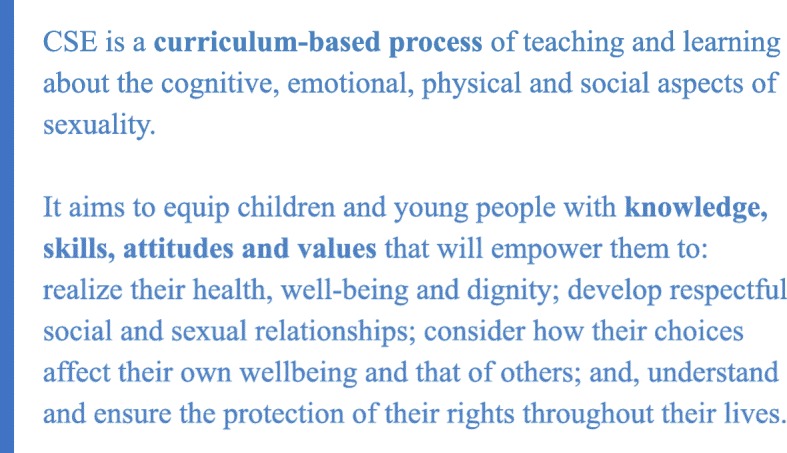
Definition of CSE, as established in the revised Guidance

The revised Guidance builds on the original Guidance, with improvements and updates based on new evidence and good practice documented from across the globe. User-surveys and structured consultations with representatives from a wide range of fields and interest-groups informed and guided the revision process, enabling the UN partners to explore how the concept of CSE has evolved with time and thereby reflect this consensus in the updated topics and learning objectives. The development of one, commonly agreed definition of the term 'CSE' was a major milestone in this process (Fig. 1).

**Fig. 2:**
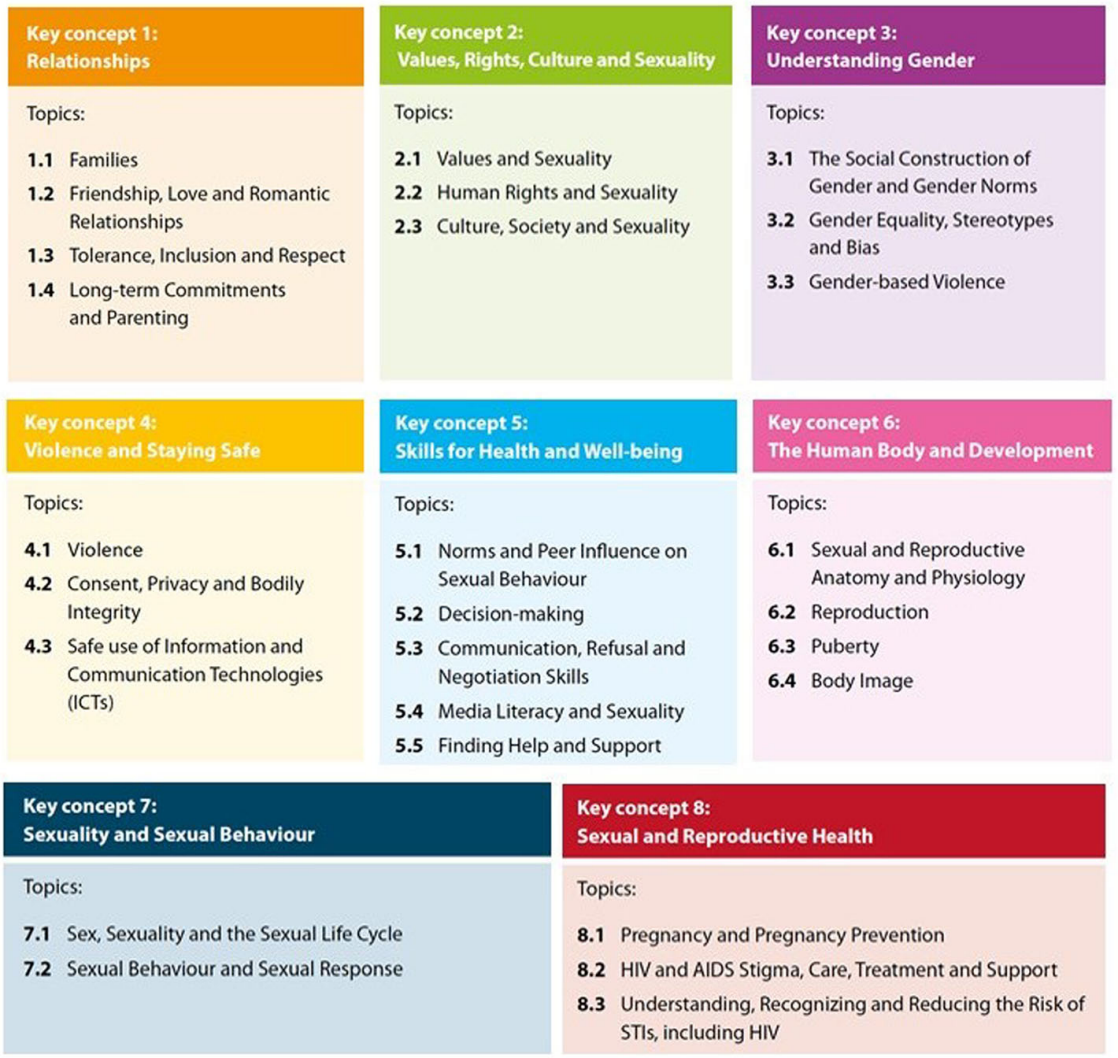
Overview of the key concepts, topics and learning objectives included in the revised Guidance

First, while the original Guidance positioned CSE in the context of the HIV response, evidence and practice have provided a deeper understanding of the immense relevance of CSE to children and adolescents’ healthy development and overall well being. In response, the revised Guidance enhances and expands its key concepts, topics and learning objectives to include issues such as early pregnancy, unsafe abortion, and gender-based violence, along with their prevention (Fig. 2). It also includes new content areas such as safe and responsible use of the Internet and social media; tolerance, inclusion, and respect; and pleasure and enjoyment of one’s sexuality.

**Fig. 3:**
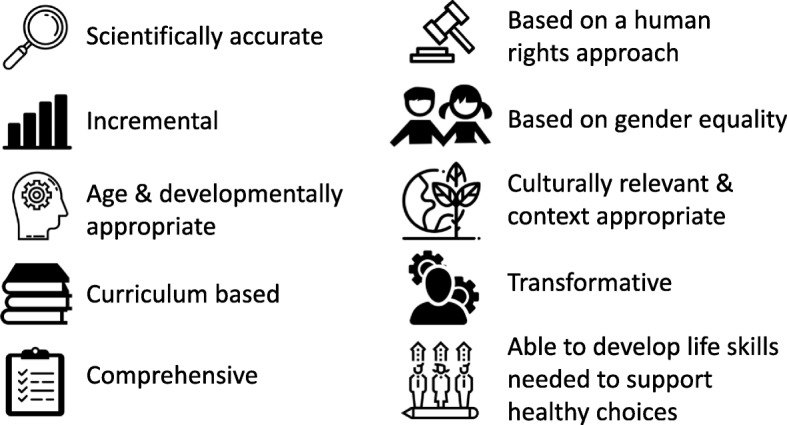
Key features of CSE, as defined in the revised Guidance

Second, with recognition that CSE can and should go beyond promoting individual knowledge and building life skills, the revised Guidance places a stronger focus on gender. This focus enables learners to explore the influence of gender inequality and gender norms on their understanding of themselves, their values, and their ability to make choices which impact their health. It also conveys CSE’s firm grounding in human rights and a reflection of the broad concept of sexuality as a natural part of human development, promoting structured learning about sex and relationships in a manner that is positive, affirming, and centered on the best interest of the young person (Fig. 3).

Third, with recognition that progress at implementing CSE in many countries and geographical regions has been slow and that deep-seated discomfort about adolescent sexuality persists, the revised Guidance provides guidance on building support and planning for the implementation of CSE programmes [7]. Recalling the evidence that sexuality education has the most impact when school-based programmes are complemented by youth –friendly health services and integrate the involvement of parents and communities, the revised Guidance reviews strategies for using such evidence to demonstrate young people’s existing needs within national and local contexts. It also explores international, regional and local frameworks and agreements that can be used to support implementation of CSE at different levels.

Lastly, the revised Guidance reflects the contribution of CSE to the realization of multiple SDGs, in particular those aimed to achieve good health and well being (Goal 3), quality education (Goal 4), and gender equality (Goal 5).

## How will the revised Guidance support CSE implementation?

Countries are encouraged but not obliged to use the revised Guidance to design and deliver CSE. UN co-publishers and their partners will promote the use of the revised Guidance within existing education and health sector efforts to strengthen and scale-up national programmes. This includes continued attention to key education inputs such as curriculum review and revision, teacher training and monitoring and evaluation of programme delivery, all aimed at ensuring that all learners benefit from CSE of good quality. The revised Guidance will also be positioned as a key resource to inform international policy, funding and practice and to leverage innovative opportunities and partnerships. UNESCO and its partners will support regeneration of educational communities of practice and promote the professionalizing of CSE, as well as encourage a positive interface between CSE, newer technologies and non-traditional learning spaces. Aligning national programmes to the content recommended in this revised Guidance will take time and concerted support. Moreover, ensuring quality and fidelity in the delivery of CSE will remain the major imperative for all concerned partners.

## Why is the revised Guidance particularly relevant today?

Now, more than ever, children and adolescents want and need information and skills that enable them to thrive as they transition to adulthood. Similarly, the SDGs and other current health and development agendas provide a platform for advocacy and a mechanism for operationalizing CSE to improve the SRH of adolescents and young people. The revised Guidance, with its unified voice, progressive position, and attention to key implementation challenges, is a responsive, timely, and critically needed tool to advance towards a tipping point for CSE.

## Abbreviations

CSE: Comprehensive sexuality education
HIV: Human immunodeficiency virus
SDG: Sustainable Development Goals
SRH: Sexual and reproductive health
UN: United Nations
UNAIDS: Joint United Nations Programme on HIV/AIDS
UNESCO: United Nations Educational, Scientific and Cultural Organization
UNFPA: United Nations Population Fund
UNICEF: United Nations Children’s Fund
WHO: World Health Organization
