# Correction to: the revised international technical guidance on sexuality education - a powerful tool at an important crossroads for sexuality education

**DOI:** 10.1186/s12978-019-0675-z

**Published:** 2019-03-11

**Authors:** Joanna Herat, Marina Plesons, Chris Castle, Jenelle Babb, Venkatraman Chandra-Mouli

**Affiliations:** 1 UNESCO Section of Health and Education, Paris, France; 2 WHO Department of Reproductive Health and Research/Human Reproduction Programme, Geneva, Switzerland

**Correction to: Reproductive Health (2018) 15:185**

**https://doi.org/10.1186/s12978-018-0629-x**

In the version of this article that was originally published [1]; foreign language translations were not included. This has now been corrected and the translations have been added in addition to the original English work. These translations are included below (see Additional files 1 and 2).

## Additional files

Additional file 1: French translations (PDF 725 kb)

Additional file 2: Spanish translations (PDF 332 kb)
