# Supplementary material for: Correction to: the revised international technical guidance on sexuality education - a powerful tool at an important crossroads for sexuality education
Source: Reprod Health. 2019 Mar 11;16:30. doi: 10.1186/s12978-019-0675-z (PMC6410497; doi:10.1186/s12978-019-0675-z)
Supplement: Supplementary file 1 — French translations (PDF 725 kb) [file 12978_2019_675_MOESM1_ESM.pdf]

Edition révisée des Principes directeurs internationaux sur l'éducation sexuelle - Un outil puissant à la croisée des chemins de l'éducation sexuelle

Joanna Herat [†], Marina Plesons[†*], Chris Castle, Jenelle Babb, Venkatraman Chandra-Mouli

Joanna Herat
Section de la santé et de l'éducation de l'UNESCO
Paris, France
j.herat@unesco.org

Marina Plesons
Département Santé et recherche génésiques de l'OMS/ Programme relatif à la reproduction humaine
Genève, Suisse
plesonsm@who.int

Chris Castle
Section de la santé et de l'éducation de l'UNESCO
Paris, France
C.Castle@unesco.org

Jenelle Babb
Section de la santé et de l'éducation de l'UNESCO
Paris, France
j.babb@unesco.org

Venkatraman Chandra-Mouli
Département Santé et recherche génésiques de l'OMS/ Programme relatif à la reproduction humaine
Genève, Suisse
chandramouliv@who.int

[†] Contributeurs à parts égales
[*] Auteur principal

Abstrait

En janvier 2018, l'UNESCO a achevé, conjointement avec l'ONUSIDA, le FNUAP, l'UNICEF, ONU-Femmes et l'OMS, le processus technique et politique substantiel d'actualisation des Principes directeurs internationaux sur l'éducation à la sexualité. Ce processus a ainsi permis d'unifier la position des Nations Unies sur le fondement, les bases factuelles et les lignes directrices quant à la conception et la dispense d'une éducation sexuelle complète (ESC). S'appuyant sur les lignes directrices initiales, le guide révisé comporte des améliorations et des mises à jour basées sur les nouvelles preuves et les bonnes pratiques recensées à travers le monde. Le processus d'actualisation été éclairé et guidé par les résultats d'enquêtes menées auprès d'usagers ainsi que par des consultations structurées avec des représentants d'un large éventail de domaines et de groupes d'intérêts. L'Edition révisée des principes directeurs présente une définition commune de l'ESC; améliore et élargit ses concepts clés, ses thèmes et ses objectifs d'apprentissage; met davantage l'accent sur le genre et les droits de l'homme; fournit des conseils sur la mobilisation d'un soutien en faveur de la mise en œuvre d'un programmes d'ESC et sa planification; et illustre la contribution qu'apporte l'ESC à la réalisation de multiples objectifs de développement durable. En raison de la voix unifiée que présente le guide révisé, sa position progressive et l'attention qu'il porte aux principaux problèmes de mise en œuvre d'une ESC, il constitue un outil réactif, opportun et indispensable pour progresser vers un point de basculement pour l'application à grande échelle d'une ESC de qualité.

Mots clés

Éducation sexuelle complète ; adolescents ; santé sexuelle et génésique

En janvier 2018, l'UNESCO a achevé, conjointement avec ONUSIDA, le FNUAP, UNICEF, ONU Femmes et l'OMS, le processus technique et politique substantiel d'actualisation des Principes directeurs internationaux sur l'éducation à la sexualité. Ce processus a ainsi permis d'unifier la position des Nations Unies sur le fondement, les bases factuelles et les lignes directrices pour la conception et la dispense d'une éducation sexuelle complète (ESC). [1] Cela a eu des conséquences considérables pour l'avancement des programmes de développement mondiaux ainsi que pour la santé et le bien-être des adolescents du monde entier.

Pourquoi les principes directeurs ont-ils été actualisés ?

Depuis la publication initiale des Principes Directeurs en 2009, le domaine de l'ESC a rapidement évolué à la lumière des résultats de la recherche et des enseignements tirés de la mise en œuvre de programmes d'éducation sexuelle dans divers contextes éducatifs [2].

Les preuves demeurent convaincantes quant à la pertinence de l'ESC pour les connaissances et les comportements des enfants et des adolescents en matière de santé et de bien-être. Dans certaines parties du monde, deux filles sur trois ont déclaré n'avoir aucune idée de ce qui leur arrivait lorsqu'elles ont commencé à avoir leurs règles. [3] L'année précédente, il a été reporté que dans un certain nombre de pays, moins de 50% des jeunes de 15 à 24 ans ont utilisé un préservatif lors du dernier rapport sexuel à haut risque. [4] Dans de trop

> ESC est un processus d'enseignement et d'apprentissage **basé sur un programme d'étude** qui traite des aspects cognitifs, émotionnels, physiques et sociaux de la sexualité.
>
> Il vise à doter les enfants et les jeunes **de connaissances, de compétences, d'attitudes et de valeurs** qui leur permettront de: réaliser leur objectifs en matière de santé, de bien-être et de dignité; développer des relations sociales et sexuelles respectueuses; examiner comment leurs choix affectent leur propre bien-être et celui des autres; et, comprendre et assurer la protection de leurs droits tout au long de leur vie.

Figure 1 Définition de l'éducation sexuelle complète, telle qu'énoncée dans l'Edition révisée des principes directeurs.

nombreuses régions du monde, les problèmes de santé sexuelle et reproductive (SSR), tels que ceux-mentionnés ci-dessus, empêchent de nombreux apprenants, en particulier les filles, d'exercer leur droit à l'éducation. Les problèmes de santé associés qui en résultent aggravent davantage la situation et ont des effets néfastes sur les opportunités de vie et le potentiel des jeunes.

Entre-temps, les données scientifiques quant à la pertinence de l'ESC pour les connaissances et les comportements des enfants et des adolescents en matière de santé et de bien-être se sont multipliées. À l'échelle mondiale, près de 50% des filles âgées de 15 à 19 ans pensent qu'un mari ou un partenaire est en droit de frapper ou de battre sa femme (ou sa partenaire) dans les circonstances suivantes : si la femme se dispute avec son mari, si elle sort sans lui dire, si elle néglige ses enfants, si elle refuse d'avoir des relations sexuelles avec lui ou si elle fait brûler le repas [5]. Si l'ESC contribue significativement à la construction de sociétés inclusives et durables, elle apporte une valeur significative lorsque les problèmes de SSR sont façonnés par des attitudes et des normes sociales et culturelles qui perpétuent les inégalités de genre et de pouvoir.

L'ESC a donc été reconnue comme un point d'entrée important pour la promotion de la santé des adolescents qui a acquis une priorité mondiale grâce aux objectifs de développement durable (ODD). Les ODD accordent une attention accrue aux adolescents, ceci à la fois comme une fin en soi et comme un moyen de réaliser la santé et le bien-être général des populations. En outre, les programmes et cadres mondiaux pour la santé, l'éducation et le développement, en particulier le programme de développement durable à l'horizon 2030, ont évolué vers une reconnaissance accrue des liens intrinsèques entre l'éducation, la santé et le bien-être, l'égalité des genres et les droits de l'homme.

Qu'y a-t-il de nouveau dans l'édition révisée des principes directeurs ?

S'appuyant sur les lignes directrices initiales, l'Edition révisée comporte des améliorations et des mises à jour basées sur les nouvelles preuves et les bonnes pratiques recensées à travers le monde. Le processus d'actualisation a été éclairé et guidé par les résultats d'enquêtes menées auprès d'usagers ainsi que par des consultations structurées avec des représentants d'un large éventail de domaines et de groupes d'intérêts. Ceci a permis aux partenaires des Nations Unies d'explorer comment le concept de l'ESC a évolué au fil du temps et ainsi de refléter ce consensus dans les nouveaux thèmes et objectifs d'apprentissage. L'élaboration d'une définition commune de l'ESC a été une étape importante dans ce processus.

Premièrement, alors que l'édition initiale des principes directeurs plaçait l'ESC dans le contexte de la lutte contre le VIH, les données scientifiques et la pratique ont permis de mieux comprendre sa pertinence considérable pour le développement sain et le bien-être général des enfants et des adolescents. En conséquence, l'édition révisée du ITGSE comporte une amélioration et un élargissement des concepts clés, des thèmes et des objectifs d'apprentissage afin d'inclure des

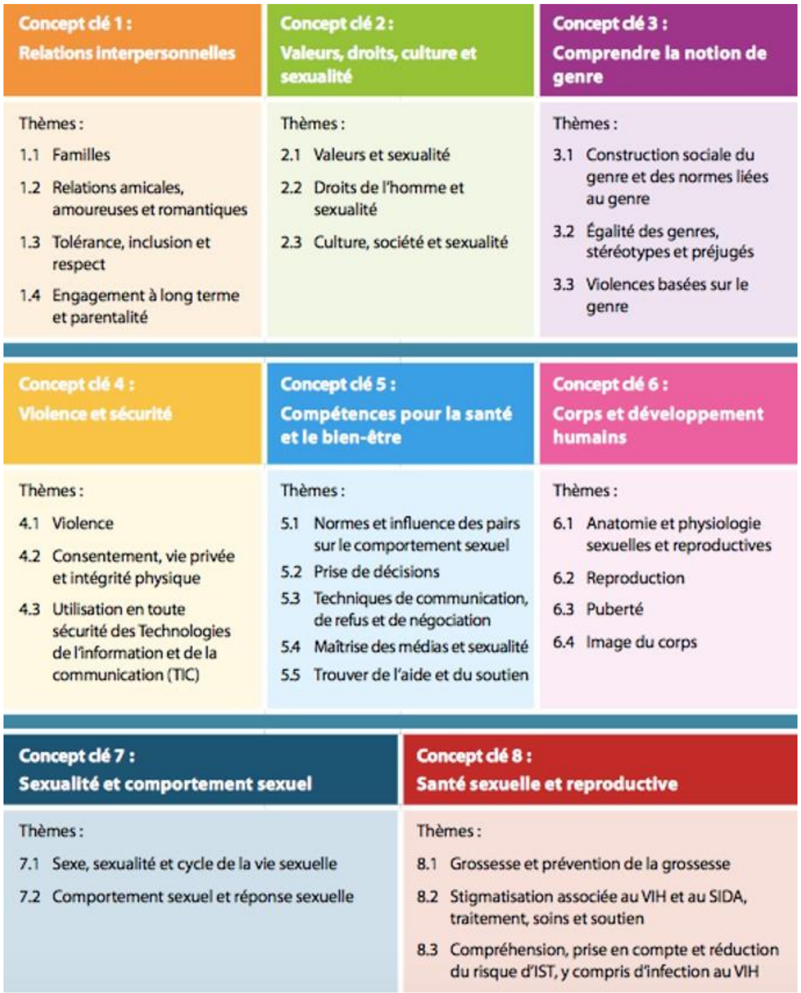

Figure 2. Aperçu des concepts clés, des sujets et des objectifs d'apprentissage inclus dans l'édition révisée des directives.

problèmes tels que la grossesse précoce, l'avortement à risque et la violence sexiste, ainsi que leur prévention. Elle inclut également de nouveaux domaines tels que l’utilisation sûre et responsable de l’Internet et des médias sociaux ; la tolérance, l’inclusion et le respect ; et le plaisir et l’épanouissement sexuel.

Deuxièmement, reconnaissant que l’ESC peut et doit dépasser la promotion des connaissances individuelles et du développement des compétences de vie, l'édition révisée met davantage l'accent sur le genre. Cette approche permet aux apprenants d'explorer comment l'inégalité de genre et des normes de genre influence la compréhension qu’ils ont d'eux-mêmes, de leurs valeurs et de leur capacité à faire des choix qui impactent leur santé. Cette édition témoigne également des bases solides de l’ESC dans le domaine des droits de la personne et du concept général de la sexualité en tant que composante naturelle du développement humain. Elle promeut un apprentissage structuré de la sexualité et des rapports entre hommes et femmes d’une manière positive, affirmée et centrée sur le meilleur intérêt d’une jeune personne.

Troisièmement, reconnaissant que les progrès réalisés dans de nombreux pays et régions géographiques, en matière de mise en œuvre de l'ESC, ont été lents et que l'inconfort profondément ancré au sujet de la sexualité des adolescents persiste, l'édition révisée fournit des conseils sur la mobilisation d'un soutien en faveur de la mise en œuvre d’un programme d’ESC et sa planification. Rappelant les

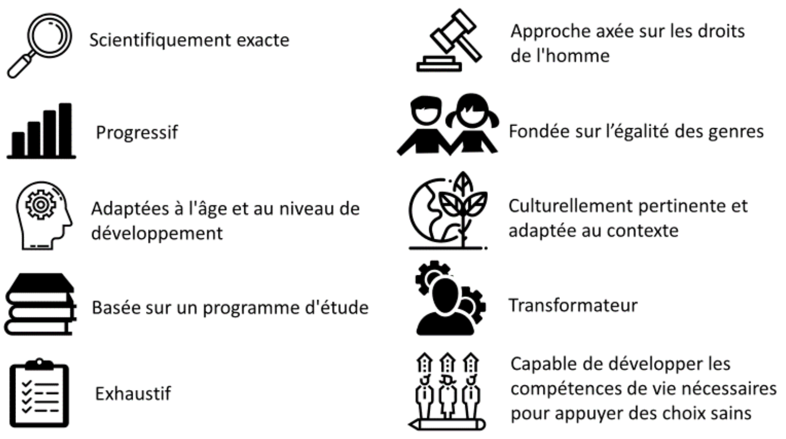

Figure 3. Principales caractéristiques de l’éducation sexuelle complète, telles que définies dans l’édition révisée des principes directeurs.

preuves que l'éducation sexuelle a le plus d'impact lorsque les programmes dispensés en milieu scolaire sont complétés par des services de santé adaptés aux jeunes, et qu'ils intègrent la participation des parents et des communautés, les lignes directives révisées examinent les stratégies permettant d'utiliser ces données probantes pour démontrer les besoins existants des jeunes dans les contextes nationaux / locaux. Elles explorent également les cadres et accords internationaux, régionaux et locaux qui peuvent être utilisés pour soutenir la mise en œuvre de l'ESC à différents niveaux.

Enfin, l'édition révisée reflète la contribution de l'ESC à la réalisation de plusieurs objectifs de développement durable, en particulier ceux qui visent à assurer la santé et le bien-être (objectif 3), une éducation de qualité (objectif 4) et l'égalité des genres (objectif 5).

Comment les principes directeurs révisés soutiendront-ils la mise en œuvre de l'ESC?

Les pays sont invités, mais non contraints, à utiliser les lignes directives révisées pour concevoir et mettre en œuvre l'ESC. Les coéditeurs onusiens et leurs partenaires encourageront l'utilisation des nouvelles directives dans le cadre des efforts déployés pour renforcer et développer les programmes nationaux dans le secteur de l'éducation et de la santé. Cela inclut une attention soutenue aux intrants clés de l'éducation tels que la révision du programme d'enseignement, la formation des enseignants et le suivi et l'évaluation de l'exécution du programme. Tous visant à garantir que tous les apprenants puissent bénéficier d'une ESC de bonne qualité. Les directives révisées seront également considérées comme une ressource clé pour éclairer les politiques, le financement et les pratiques internationales et pour tirer parti d'opportunités novatrices et de partenariats. L'UNESCO et ses partenaires soutiendront la régénération des communautés éducatives et encourageront la professionnalisation de l'ESC, tout en encourageant une interface

positive entre l’ESC, les nouvelles technologies et les espaces d'apprentissage non traditionnels. Aligner les programmes nationaux sur le contenu recommandé dans cette édition révisée prendra du temps et nécessitera un appui concerté. Par ailleurs, l’impératif majeur pour tous les partenaires concernés demeurera de garantir la qualité et la fidélité de la mise en œuvre de l’ESC.

Pourquoi les principes directeurs révisés sont-ils particulièrement pertinents aujourd'hui ?

Aujourd'hui plus que jamais, les enfants et les adolescents veulent et ont besoin d'informations et de compétences qui leur permettent de s'épanouir au cours de leur transition vers l'âge adulte. De même, les ODD et autres programmes actuels en matière de santé et de développement fournissent un cadre aux activités de plaidoyer et un mécanisme d’opérationnalisation de l’ESC afin d'améliorer la santé sexuelle et reproductive des adolescents et des jeunes. En raison de la voix unifiée que présente le guide révisé, sa position progressive et l’attention qu’il porte aux principaux problèmes de mise en œuvre d’une ESC, il constitue un outil réactif, opportun et indispensable pour progresser vers un point de basculement pour l'application à grande échelle d’une ESC de qualité.

Déclarations

Approbation éthique et consentement à participer

Non-applicable

Consentement à la publication

Non-applicable

Disponibilité des données et du matériel

Non-applicable

Conflits d’intérêts

Les auteurs déclarent ne pas avoir d’intérêts concurrents.

Financement

Non-applicable

Authors' contributions

VCM a conçu le document. MP a préparé l’avant-projet du document avec l’aide de JH et l’a partagé avec les coauteurs. CC, JH, JB et VCM ont examiné le projet et fourni leurs contributions. MP a révisé le projet. Tous les auteurs ont lu et approuvé le manuscrit final.

Remerciements

Non-applicable

Abréviations

ESC	Education sexuelle complète
VIH	Virus de l'immunodéficience humaine
ITGSE	Principes directeurs internationaux sur l'éducation à la sexualité
ODD	Objectifs de développement durable
SSR	Santé sexuelle et and reproductive
NU	Nations Unies
ONUSIDA	Programme commun des Nations Unies sur le VIH/sida
FNUAP	Fonds des Nations unies pour la population
UNESCO	Organisation des Nations Unies pour l'éducation, la science et la culture
UNICEF	Le Fonds des Nations Unies pour l'enfance
OMS	Organisation Mondiale de la Santé

Références

1. UNESCO et al. Edition révisée des Principes directeurs internationaux sur l'éducation à la sexualité. Paris: UNESCO; 2018. Disponible à l'adresse suivante : < http://unesdoc.unesco.org/images/0026/002608/260840f.pdf >. Consultée le 29 août 2018.
2. UNESCO. VIH et éducation sexuelle complète. Paris: UNESCO; 2018. Disponible à l'adresse suivante : <https://en.unesco.org/themes/health-education/hiv-sexuality-education> . Consultée le 29 août 2018.
3. Dasra, Kiawah Trust, et l'USAID. Lumière sur : Améliorer la santé et l'hygiène menstruelles en Inde. Rapport. Mumbai: Dasra: 2014. Disponible à l'adresse suivante : <http://menstrualhygieneday.org/wp-content/uploads/2017/03/Spot-On.pdf>. Consultée le 29 août 2018.
4. ONUSIDA. Mettre fin au sida, progrès vers les objectifs 90-90-90, Le point sur le sida dans le monde. Genève: ONUSIDA;2017. Disponible à l'adresse suivante : < http://www.unaids.org/en/resources/documents/2017/20170720_Global_AIDS_update_2017 >. Consultée le 29 août 2018.
5. UNICEF. Data d'UNICEF: Attitudes et normes sociales sur la violence. New York: UNICEF;2017. Disponible à l'adresse suivante : < https://data.unicef.org/topic/child-protection/violence/attitudes-and-social-norms-on-violence/>. Consultée le 29 août 2018.
6. UNESCO. Éducation sexuelle complète : Les défis et les opportunités de passer à l'échelle supérieure. Paris : UNESCO; 2014. Disponible à l'adresse suivante : <http://unesdoc.unesco.org/images/0022/002277/227781e.pdf>. Consultée le 29 août 2018.
