# Supplementary material for: Correction to: the revised international technical guidance on sexuality education - a powerful tool at an important crossroads for sexuality education
Source: Reprod Health. 2019 Mar 11;16:30. doi: 10.1186/s12978-019-0675-z (PMC6410497; doi:10.1186/s12978-019-0675-z)
Supplement: Supplementary file 2 — Spanish translations (PDF 332 kb) [file 12978_2019_675_MOESM2_ESM.pdf]

Educación integral en sexualidad- una pieza que falta en la salud de los adolescentes

Joanna Herat †, Marina Plesons † *, Chris Castle, Jenelle Babb, Venkatraman Chandra-Mouli

Joanna herat
Sección de Salud y Educación de la UNESCO
París, Francia
j.herat@unesco.org

Marina Plesons
Departamento de Salud Reproductiva e Investigación de la OMS / Programa de Reproducción Humana
Ginebra, Suiza
plesonsm@who.int

Chris Castle
Sección de Salud y Educación de la UNESCO
París, Francia
C.Castle@unesco.org

Jenelle babb
Sección de Salud y Educación de la UNESCO
París, Francia
j.babb@unesco.org

Venkatraman Chandra-Mouli
Departamento de Salud Reproductiva e Investigación de la OMS / Programa de Reproducción Humana
Ginebra, Suiza
chandramouliv@who.int

† Contribuyentes por igual
* Autor principal

Resumen

En enero de 2018, la UNESCO, junto con ONUSIDA, UNFPA, UNICEF, ONU Mujeres y la OMS completó el sustancial proceso técnico y político de actualización de las Orientaciones técnicas internacionales sobre educación en sexualidad, unificando así una posición de la ONU sobre la justificación, la evidencia y la orientación sobre el diseño e implementación de la educación integral en sexualidad (EIS).

Las Orientaciones revisadas se basan en las Orientaciones originales, con mejoras y actualizaciones basadas en nuevas evidencias y buenas prácticas documentadas en todo el mundo. Encuestas de usuarios y consultas estructuradas con representantes de una amplia gama de campos y grupos de interés informaron y guiaron el proceso de revisión. Las Orientaciones revisadas presentan una definición consensuada de la EIS; mejora y expande sus conceptos clave, temas y objetivos de aprendizaje; pone un foco fortalecido en género y derechos humanos; proporciona orientación sobre la construcción de soporte y planificación para la implementación de programas de EIS; y refleja la contribución de la EIS a la realización de múltiples ODS. Con su voz unificada, posición progresiva y atención a los principales desafíos de implementación, las Orientaciones revisadas son una herramienta sensible, oportuna y críticamente necesaria para avanzar hacia un punto de inflexión para la aplicación a gran escala de EIS de calidad.

Palabras clave

Educación integral en sexualidad; adolescentes; salud sexual y reproductiva

En enero de 2018, la UNESCO, junto con ONUSIDA, UNFPA, UNICEF, ONU Mujeres y la OMS completó el importante proceso técnico y político de actualización de la las Orientaciones

técnicas internacionales sobre educación en sexualidad, unificando así una posición de la ONU sobre la justificación, la evidencia y la orientación sobre el diseño y la educación integral en sexualidad (EIS). [1] Este logro tiene repercusiones rotundas para el avance de las agendas de desarrollo global y para la salud y el bienestar de los adolescentes en todo el mundo.

¿Por qué se actualizaron las Orientaciones?

Desde que se publicaron las Orientaciones originales en 2009, el campo de la EIS ha evolucionado rápidamente a la luz de evidencia proporcionada por investigaciones y lecciones aprendidas de la implementación de programas de educación en sexualidad en diversos entornos educativos. [2]

Evidencia de la relevancia de la EIS para los conocimientos y conductas de niños y adolescentes relacionados con su salud y bienestar continúa siendo convincente. En algunas partes del mundo, dos de cada tres niñas informaron que no tenían idea de lo que les estaba pasando cuando comenzaron a menstruar. [3] En varios países, el uso de condones en la última relación sexual de alto riesgo del año anterior fue menos del 50% para jóvenes de 15-24 años. [4] En demasiados lugares del mundo, las cuestiones y los problemas de salud sexual y reproductiva (SSR) como estos, son barreras para que muchos estudiantes- especialmente las

> La EIS es un **proceso de enseñanza y aprendizaje basado en planes de estudios** que versa sobre los aspectos cognitivos, psicológicos, físicos y sociales de la sexualidad.
>
> Su propósito es dotar a los niños y jóvenes de **conocimientos basados en datos empíricos, habilidades, actitudes y valores** que los empoderarán para disfrutar de salud, bienestar y dignidad; entablar relaciones sociales y sexuales basadas en el respeto; analizar cómo sus decisiones afectan su propio bienestar y el de otras personas; y comprender cómo proteger sus derechos a lo largo de su vida y velar por ellos.

Figure 1. Definición de educación integral en sexualidad, según lo establecido en las Orientaciones revisadas.

niñas- puedan cumplir con su derecho a la educación. Los pobres resultados en materia de salud asociados se combinan y afectan negativamente sus oportunidades y potencial de vida.

Mientras tanto, la evidencia de la relevancia de la EIS para las actitudes y las normas relacionadas con su salud y bienestar de los niños y adolescentes se ha fortalecido. En todo el mundo, cerca del 50% de todas las niñas de entre 15 y 19 años de edad creen que un marido o una pareja se encuentra justificado para golpear a su esposa (o pareja) en ciertas circunstancias - si la esposa discute con su esposo, sale sin decirle, descuida a los niños, se niega a tener relaciones sexuales con él o quema la comida. [5]

Cuando las cuestiones de SSR están determinadas por actitudes y normas sociales y culturales que afianzan las desigualdades de género y poder, la función transformadora de la EIS para desafiar tales actitudes y normas agrega valor a la ya importante contribución del EIS en la construcción de sociedades inclusivas y sostenibles.

La EIS ha sido reconocido como un importante punto de entrada para la promoción de la salud de los adolescentes, lo cual ha ganado prioridad mundial a través de los Objetivos de Desarrollo Sostenible (ODS) que piden atención para los adolescentes como un fin en sí mismo y como un medio para el fin de la salud en general y bienestar de las poblaciones. Además, los programas y marcos mundiales de salud, educación y desarrollo, en particular la Agenda 2030 para el desarrollo sostenible, se han orientado hacia un mayor reconocimiento de los vínculos intrínsecos entre educación, salud y bienestar, igualdad de género y derechos humanos.

¿Qué hay de nuevo en las Orientaciones revisadas?

La edición revisada de las OTIES se basa en las Orientaciones originales, con mejoras y actualizaciones basadas en nuevas evidencias y buenas prácticas documentadas en todo el mundo. Encuestas a usuarios y consultas estructuradas con representantes de una amplia gama de campos y grupos de interés informaron y guiaron el proceso de revisión, permitiendo a los socios de la ONU explorar cómo el concepto de la EIS ha evolucionado con el tiempo, y de este modo reflejar este consenso en los temas actualizados y objetivos de aprendizaje. El desarrollo de una definición consensuada de este término fue un hito importante en este proceso.

En primer lugar, si bien las Orientaciones originales posicionaron a la EIS en el contexto de la respuesta al VIH, la evidencia y la práctica han proporcionado una comprensión más profunda de la inmensa relevancia de la EIS para el desarrollo saludable y el bienestar general de los niños y adolescentes.

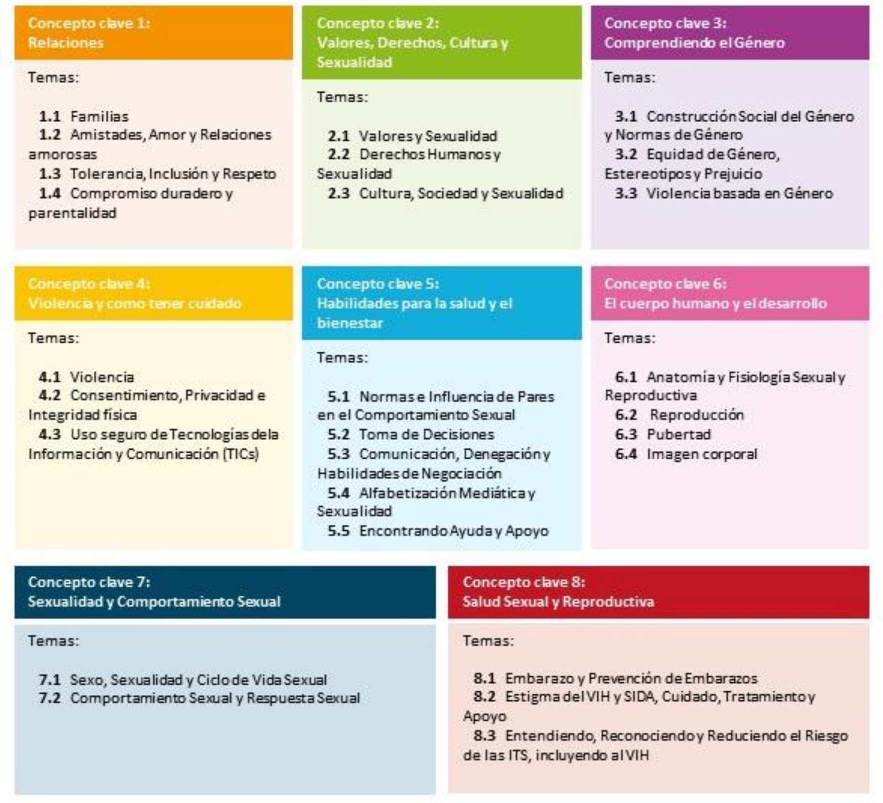

Figure 2. Descripción general de los conceptos clave, temas y objetivos de aprendizaje incluidos en las Orientaciones revisadas.

En respuesta, la edición revisada de las OTIES mejora y amplía sus conceptos clave, temas y objetivos de aprendizaje para incluir cuestiones como el embarazo precoz, el aborto inseguro y la violencia de género, así como su prevención. También incluye nuevas áreas de contenido tales

como el uso seguro y responsable de Internet y las redes sociales; tolerancia, inclusión y respeto; y placer y disfrute de la propia sexualidad.

En segundo lugar, reconociendo que la EIS puede y debe ir más allá de promover el conocimiento individual y la construcción de habilidades para la vida, la edición revisada pone mayor énfasis en el género. Este enfoque permite a los estudiantes explorar la influencia de las desigualdades de género y las normas de género en su comprensión de sí mismos, sus valores y su capacidad para tomar decisiones que afectan su salud. También transmite bases sólidas de la EIS en derechos humanos y un reflejo del amplio concepto de la sexualidad como parte natural del desarrollo humano, promoviendo el aprendizaje estructurado sobre el sexo y las relaciones de una manera positiva, afirmativa y centrada en el mejor interés de la persona joven.

En tercer lugar, reconociendo que el progreso en la implementación de la EIS en muchos países y regiones geográficas ha sido lento y la persistencia de una incomodidad profundamente arraigada sobre la sexualidad de los adolescentes, la edición actual ofrece orientación sobre la creación de apoyo y planificación para la implementación de programas de EIS. [6] Recordando la evidencia de que la educación en sexualidad tiene el mayor impacto cuando los programas escolares se complementan con servicios de salud amigables para los jóvenes e integran la participación de padres y comunidades, en las Orientaciones revisadas se revisan las estrategias para usar dicha evidencia para demostrar las necesidades existentes de los jóvenes

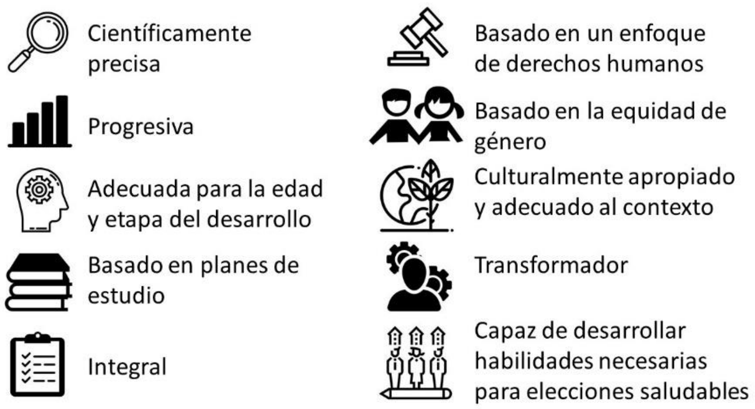

Figure 3. Características clave de la educación integral en sexualidad, tal como se define en las Orientaciones revisadas.

dentro de contextos nacionales/locales. También explora marcos y acuerdos internacionales, regionales y locales que pueden utilizarse para apoyar la implementación de la EIS en diferentes niveles.

Por último, la edición revisada refleja la contribución de la EIS a la realización de múltiples ODS, en particular aquellos destinados a lograr una buena salud y bienestar (Objetivo 3), educación de calidad (Objetivo 4) e igualdad de género (Objetivo 5).

¿Cómo apoyarán las Orientaciones revisadas a la implementación de la EIS?

Se alienta a los países, pero no se les obliga, a utilizar las Orientaciones revisadas para diseñar e implementar la EIS. Los coeditores de las Naciones Unidas y sus socios promoverán el uso de as Orientaciones revisadas dentro de los esfuerzos existentes de los sectores de educación y salud para fortalecer y ampliar los programas nacionales. Esto incluye la atención continuada a insumos claves de la educación, como la revisión y actualización de la currícula, la capacitación de docentes y el seguimiento y la evaluación de la ejecución de los programas, todo dirigido a garantizar que todos los alumnos se beneficien de EIS de buena calidad. Las Orientaciones revisadas también se posicionarán como un recurso clave para informar la política internacional, la financiación y la práctica, así como para aprovechar oportunidades y asociaciones innovadoras. La UNESCO y sus socios apoyarán la regeneración de comunidades educativas de práctica y promoverán la profesionalización de la EIS, así como también fomentarán una interfaz positiva entre la EIS, nuevas tecnologías y espacios de aprendizaje no tradicionales. La alineación de los programas nacionales con el contenido recomendado en estas Orientaciones revisadas llevará tiempo y un apoyo concertado. Además, garantizar la calidad y la fidelidad en

la implementación de EIS seguirá siendo el principal imperativo para todos los socios interesados.

¿Por qué las Orientaciones revisadas es particularmente relevante hoy?

Ahora, más que nunca, los niños y adolescentes quieren y necesitan información y habilidades que les permitan prosperar en su transición hacia la adultez. Del mismo modo, los ODS y otras agendas actuales de salud y desarrollo brindan una plataforma para la incidencia y un mecanismo para operacionalizar la EIS a fin de mejorar la salud sexual y reproductiva de adolescentes y jóvenes. La edición revisada de las Orientaciones Técnicas Internacionales, con su voz unificada, posición progresiva y atención a los principales desafíos de implementación, son una herramienta sensible, oportuna y críticamente necesaria para avanzar hacia un punto de inflexión para la EIS.

Declaraciones

Aprobación de ética y consentimiento a participar
No aplica

Consentimiento para publicación
No aplica.

Disponibilidad de datos y material
No aplica.

Conflicto de intereses
Los autores declaran que no tienen intereses en conflicto.

Financiación
No aplica.

Contribuciones de los autores
VCM concibió el trabajo. MP preparó el primer borrador con aportes de JH, y lo compartió con los coautores. CC, JH, JB y VCM revisaron el borrador y proporcionaron aportes. MP revisó el borrador. Todos los autores han leído y aprobado el manuscrito final.

Agradecimientos
No aplica.

Abreviaturas y Acrónimos
EIS – Educación integral en sexualidad
OTIES - Orientaciones Técnica Internacional sobre Educación en Sexualidad
ODS – Objetivos de Desarrollo Sostenible
OMS – Organización Mundial de la Salud
ONU – Organización de Naciones Unidas
ONUSIDA – Programa Conjunto de las Naciones Unidas sobre el VIH/Sida
SSR – Salud Sexual y Reproductiva
UNESCO – Organización de las Naciones Unidas para la Educación, la Ciencia y la Cultura
UNFPA – Fondo de Población de las Naciones Unidas
UNICEF – Fondo de las Naciones Unidas para la Infancia
VIH – Virus de la Inmunodeficiencia Humano

References

1. UNESCO et al. Revised edition of the International Technical Guidance on Sexuality Education. Paris: UNESCO; 2018. Available at < http://unesdoc.unesco.org/images/0026/002607/260770e.pdf>. Accessed 29 Aug 2018.
2. UNESCO. HIV and Sexuality Education. Paris: UNESCO; 2018. Available at <https://en.unesco.org/themes/health-education/hiv-sexuality-education> . Accessed 29 Aug 2018.
3. Dasra, Kiawah Trust, and USAID. Spot On! Improving Menstrual Health and Hygiene in India. Report. MumbaI: Dasra: 2014. Available at < http://menstrualhygieneday.org/wp-content/uploads/2017/03/Spot-On.pdf>. Accessed 29 Aug 2018.
4. UNAIDS. Ending AIDS, progress towards the 90-90-90 targets, Global AIDS update. Geneva: UNAIDS;2017. Available at <http://www.unaids.org/en/resources/documents/2017/20170720_Global_AIDS_update_2017>. Accessed 29 Aug 2018.
5. UNICEF. UNICEF data: Attitudes and social norms on violence. New York: UNICEF;2017. Available at < https://data.unicef.org/topic/child-protection/violence/attitudes-and-social-norms-on-violence/>. Accessed 29 Aug 2018.
6. UNESCO. Comprehensive Sexuality Education: The Challenges and Opportunities of Scaling-Up. Paris: UNESCO; 2014. Available at <http://unesdoc.unesco.org/images/0022/002277/227781e.pdf>. Accessed 29 Aug 2018.
